# Effectiveness of a proteoliposome-based vaccine against salmonid rickettsial septicaemia in *Oncorhynchus mykiss*

**DOI:** 10.1186/s13567-021-00982-2

**Published:** 2021-08-23

**Authors:** Mario Caruffo, Sonia Vidal, Leonardo Santis, Daniela Siel, Oliver Pérez, Paula R. Huenchullan, Leonardo Sáenz

**Affiliations:** 1 NGEN LAB S.A, Santiago, Chile; 2 Escuela de Biotecnología, Facultad de Ciencias, Universidad Santo Tomás, Santiago, Chile; 3 Laboratorio de Vacunas Veterinarias, Departamento de Ciencias Animales, Universidad de Chile, Santiago, Chile; 4 Instituto de Ciencias Básicas Y Preclínicas “Victoria de Girón”, Universidad de Ciencias Médicas de La Habana, Havana, Cuba

**Keywords:** Salmonid rickettsial septicemia, SRS, vaccine, proteoliposome, cell membrane, *Oncorhynchus mykiss*

## Abstract

Salmonid rickettsial septicaemia (SRS) is a contagious disease caused by *Piscirickettsia salmonis*, an intracellular bacterium. SRS causes an estimated economic loss of $700 million USD to the Chilean industry annually. Vaccination and antibiotic therapy are the primary prophylactic and control measures used against SRS. Unfortunately, commercially available SRS vaccines have not been shown to have a significant effect on reducing mortality. Most vaccines contain whole inactivated bacteria which results in decreased efficacy due to the limited ability of the vaccine to evoke a cellular mediated immune response that can eliminate the pathogen or infected cells. In addition, SRS vaccine efficacy has been evaluated primarily with *Salmo salar* (Atlantic salmon). Vaccine studies using *Oncorhynchus mykiss* (rainbow trout) are scarce, despite SRS being the leading cause of infectious death for this species. In this study, we evaluate an injectable vaccine based on *P. salmonis* proteoliposome; describing the vaccine security profile, capacity to induce specific anti-*P. salmonis* IgM and gene expression of immune markers related to T CD8 cell-mediated immunity. Efficacy was determined by experimental challenge with *P. salmonis* intraperitoneally. Our findings indicate that a *P. salmonis* proteoliposome-based vaccine is able to protect *O. mykiss* against challenge with a *P. salmonis* Chilean isolate and causes a specific antibody response*.* The transcriptional profile suggests that the vaccine is capable of inducing cellular immunity. This study provides new insights into *O. mykiss* protection and the immune response induced by a *P. salmonis* proteoliposome-based vaccine.

**Supplementary Information:**

The online version contains supplementary material available at 10.1186/s13567-021-00982-2.

## Introduction

The aquaculture industry is threatened by infectious diseases that cause severe economic losses due to low productivity. Chilean aquaculture, specifically salmon and trout farming is the largest in the world alongside Norway. Significant economic losses occur annually due to salmonid rickettsial septicaemia (SRS). SRS is a contagious disease affecting wild and cultured salmonids such as coho salmon (*Oncorhynchus kisutch*), Atlantic salmon (*Salmo salar*) and rainbow trout (*O. mykiss*) during the on-growing phase in seawater [1, 2]. The disease is caused by *Piscirickettsia salmonis* a Gram negative, facultative intracellular, nonmotile, nonencapsulated, pleomorphic but usually coccoid bacterium with an approximate diameter of 0.5–1.5 μm [3]. *P. salmonis* was originally isolated in 1989 from coho salmon in southern Chile. The disease is characterized by colonization of several organs including kidney, liver, spleen, intestine, brain, ovary, and gills [1, 4]. SRS mainly affects the Chilean industry where annual economic losses are estimated to be $ 700 million USD [3]. However, there have been reports of *P. salmonis* infections in Ireland, Norway, Canada [5, 6], and Turkey [7]. Outbreaks outside of Chile have had reduced virulence compared to those in Chile which could be the result of *P. salmonis* isolate differences and production strategies [8].

Vaccination and antibiotic therapy are the primary prophylactic and control measures used against SRS. Commercially available SRS vaccines have not significantly reduced mortality under field conditions [3]. Antibiotics have been used extensively leading to Chilean salmon farming having one of the highest rates of antibiotic consumption *per* ton of harvested fish in the world [9]; generating an economic and environmental problem. 90% of antibiotic use is for SRS treatment [10]. Antibiotic resistance is another serious problem. Therefore, the development of new curative or preventive therapies is necessary to reduce antibiotic usage and increase fish survival. The use of vaccination to prevent disease is used routinely in finfish aquaculture, especially in salmonids [11]. There are currently 56 salmonid vaccines registered in Chile; of which 34 contain *P. salmonis* antigens (SAG, December 2020) [12]. Most vaccines are administered via injection (31) with a low number of immersion (1) or oral (2) formulations. The vaccines used are mainly bacterin-based vaccines composed of *P. salmonis* whole cell inactivated with formalin or heat (30). Three of them are based on recombinant antigens and one is a live attenuated vaccine [12]. Despite the large number of available vaccines, SRS continues to be the main cause of infectious death by salmonids in Chile.

Bacterin-based vaccines are known for inducing humoral immune responses whose protective mechanism is neutralization of extracellular replicating pathogens [13]. The use of bacterin-based vaccines to immunize against extracellular bacteria has provided substantial protection against *Flavobacterium columnare*, *Vibrio anguillarum*, and *Yersinia ruckerii* [14, 15]*.* However, the efficacy of this type of vaccine against intracellular bacteria is limited due to the inability to evoke a cellular mediated immune response capable of eliminating intracellular pathogens or infected cells. The challenge for SRS vaccine development is to increase the efficacy against intracellular bacterial pathogens.

Vaccines based on bacterial proteoliposome have demonstrated an ability to induce a cellular immune response [16–20] as well as both systemic and mucosal antibody responses in mammals [21]. Proteoliposomes are made from bacterial membranes, which are solubilized through the use of detergents and cell disruption techniques such as ultrasound. Once the detergent is removed, proteoliposomes form spontaneously without damaging antigens [22]. These proteolipidic nanovesicles incorporate not only bacterial proteins but also other elements from the pathogen, such as lipopolysaccharides, bacterial cell wall or even traces of bacterial DNA, structures that have proven to have immunopotentiating and immunomodulatory effects [23]. So far, no vaccine has been developed for fish using bacterial proteoliposome as antigens. Only its use as an adjuvant has been described in an oral *A. hydrophila* bacterin adjuvanted with a proteoliposome derived microparticle that increased production of IgM in fish [22]. For these reasons, the development of a vaccine based on proteoliposomes derived from *P. salmonis* is an attractive alternative that could replace or complement other vaccines.

The aim of this work was to evaluate a new injectable vaccine formulation with proteoliposome derived from *P. salmonis* in *O. mykiss.* The vaccine was capable of inducing a specific immune response against *P. salmonis* and fish immunized with this formulation were effectively protected against a lethal *P. salmonis* challenge.

## Materials and methods

### Fish maintenance

Disease-free 40 g rainbow trout (*O. mykiss*) were obtained from a local aquaculture facility and maintained at Quillaipe experimental center (Puerto Montt, Chile). During acclimatization (14 days) and immunization (71 days) fish were maintained in 1 m^3^ tanks at a density of 11 kg/m^3^ in fresh water with an exchange rate of 0.8–1 m^3^/h. Water salinity was gradually increased to ~32 ppt (parts *per* thousand) previous to challenge. Water conditions during acclimatization and immunization were: 12 ± 1 °C and oxygen saturation was 80–110%. Fish were fed ad libitum four times a day (EWOS micro 50 and 100, EWOS, Chile).

### Preparation of vaccine formulations and vaccination

The vaccine is based on a bacterial proteoliposome generated from *P. salmonis* (LF-89, field strain) bacterial membranes. Bacteria were grown in a cell free medium, SFX-Insect (HyClone) with constant agitation for 14 days at 18 °C. Bacterial pellets were washed with phosphate buffer saline (PBS) and then frozen at −80 °C. Each gram of pellet was resuspended in 25 mL of lysis buffer (Na_2_HPO_4_ 20 mM, NaCl 0.5 M, pH 7.4 and sterilized by filtration) with 2 g of sterile zirconium silicate beads 0.1 mm (BioSpec®) and sonicated at 100% capacity (Ultrasonic Processor UP400S, Hielscher). Supernatant was recovered and centrifuged again at 20 000 *g* for 15 min. The pellet was resuspended in membrane solubilization buffer (Tris–HCl 20 mM, KCl 25 mM, Sodium deoxycholate 1.5% (w/v) at pH 10 and filtered 0.2 μm) and incubated at 100 RPM at 20 °C overnight (ON). The solution was centrifuged at 500 *g* for 5 min and supernatant was incubated in Bio-Beads (BioRad) following manufacturer instructions and resuspended in saline solution (10 mL saline/1 g pellet). The vaccine formulation was made of 10 µg of *P. salmonis* proteoliposome, emulsified with one of two adjuvants per manufacturer instructions: Montanide ISA 760 VG (vaccine 1) or ISA 763 A VG (vaccine 2) (Seppic, France) a water-in-polymer and a water-in-oil, respectively.

### Size estimation and measure of the Zeta potential

The Zeta potential of proteoliposome was measured using a Zeta potential analyzer (ZetaPlus, Brookhaven Instruments). Measurements were determined at 25 °C in an electric field of 15.62 V/cm. The size and polydispersity index were determined by light scattering using a multi-angle particle sizing option (ZetaPlus, Brookhaven Instruments). A stock solution of proteoliposome (1.5 mg/mL in ultrapure water) was used for both Zeta potential and particle size measurements. 50 µL of proteoliposome was mixed 5 mL of bi-filtered KCl (1 mM in ultrapure water; pH 6.8–7.0).

### Fish vaccination

Fish were anaesthetized with benzocaine (Kalmagin 20%, Centrovet) and vaccinated by intra-peritoneal (i.p.) injection (100 µL *per* fish, 10 µg of total protein of *P. salmonis* proteoliposome) and control groups were injected i.p. with 100 µL of sterile saline. After 300 degree-days (DD, close to 25 days) a second immunization dose was given. This *P. salmonis* proteoliposome based vaccine was developed by the Laboratory of Veterinary Vaccines, Universidad de Chile.

### Safety and adverse effects of vaccine evaluations

To evaluate vaccine safety 20 fish *per* group were sampled 300 DD post-second immunization and euthanized by overdose of benzocaine. As a measure of overall fish fitness, the weight and fork length were recorded and Fulton’s Condition Factor was calculated (K = Weight/Lenghth^3^) [24]. A post-mortem gross pathology examination of the intraperitoneal cavity was conducted and scored according to gross pathological score systems: Speilberg score according to Midtlyng et al. [25].

### Experimental design and sampling

Before the challenge experiment was performed, the median lethal dose (LD_50_) of *P. salmonis* (LF-89 type) was determined. *P. salmonis* (LF-89) was provided by ADL Diagnostic Chile Ltda. Four dilutions were assessed from a stock concentration of 1 × 10^8.5^ tissue culture infective dose 50% per mL (TCID_50_/mL, determined through the Karber-Spearman method), from which four dilutions were made by a factor of 10 (1:10 to 1:10 000). These dilutions plus a control made of L15 media (Leibovitz, Invitrogen) were administered by i.p. injection in 200 µL. Fish were distributed in five 350 L tanks (*n* = 30 fish/tank) at a density of 9 kg/m^3^ (seawater). Fish were monitored daily for 25 days and mortalities recorded. From these results the challenge dose was determined to be 1:10 000 (Additional file 1).

**Figure 1:**
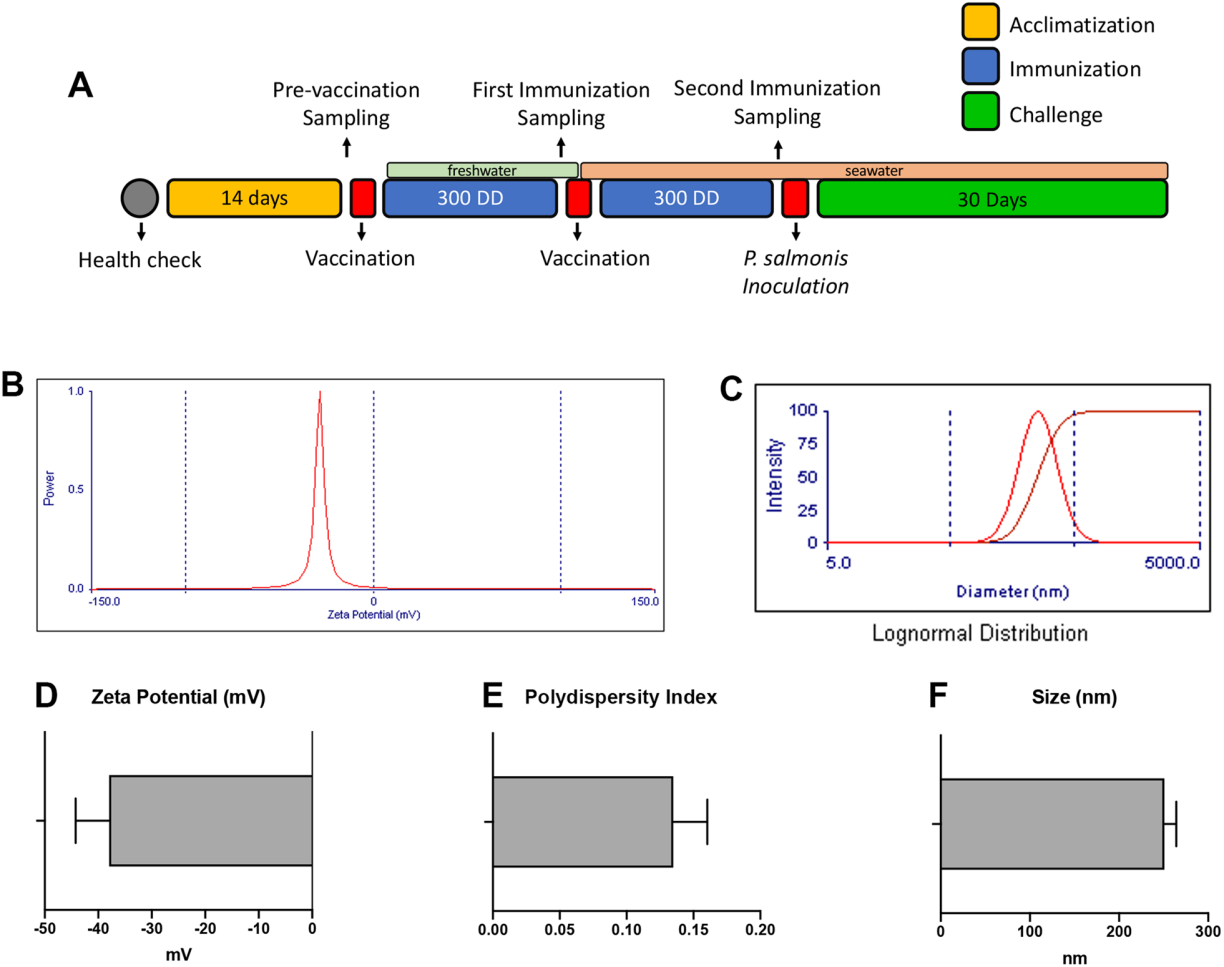
**Experimental strategy and*Piscirickettsia salmonis* proteoliposome light scattering characterization**. **A** Fish were left in acclimatization for 14 days and then were vaccinated intraperitoneally in freshwater. Salinity was increased gradually. 300 DD after first vaccination fish received a second vaccination. 300 DD later, fish were challenged with *Piscirickettsia salmonis*. Blood samples to quantify specific IgM were taken pre-vaccination, after the first immunization and second immunization (red squares). The experimental strategy can be separated into 3 steps: acclimatization (yellow), immunization (blue), challenge (green). DD, degree-days. **B** Proteoliposome zeta potential. **C** Proteoliposome size with a lognormal distribution. **D** Zeta potential shows an anionic proteoliposome with an average superficial charge of -37.9 ± 6.3 mV (*n* = 10). **E** Polydispersity index was 0.1349 ± 0.03. **F** The average diameter of proteoliposome was 250.9 ± 13.2 nm (*n* = 10).

The challenge was conducted at Quillaipe experimental center (Puerto Montt, Chile) as shown in Figure 1A. Forty fish *per* condition (vaccine 1, vaccine 2 and control) were stocked in 720 L tanks at a density of 24 kg/m^3^ in seawater. Fish from each treatment group were in each tank. All fish were marked with a PIT tag (Passive Integrated Transponder, ID-100 microtransponder, Trovan) to identify treatment groups for sampling and mortality analysis. Bacterial challenge was performed in triplicate by intraperitoneal inoculation.

Five fish *per* treatment were anesthetized for sampling in pre-vaccination, after the first immunization and after the second immunization (Figure 1A). Blood samples were taken from the caudal vein with a 3 mL syringe and stored at 4 °C for 24 h and then centrifuged for 10 min at 6000 *g*. Serum samples were stored at -20 °C until use to evaluate antibody production. After blood samples fish were euthanized by overdose of benzocaine and anterior kidney samples were taken to address gene expression. Sampling was performed just previous to experimental challenge to avoid additional stress during the course of the challenge. Mortality was recorded daily until day 25 post-challenge and confirmed by qPCR analysis according to Karatas et al. [26].

### Antibody ELISA

Nunc Polysorb plates were activated with 40 µg/mL of total protein extracts from *P. salmonis* (LF-89, field strain) in bicarbonate buffer (NaHCO_3_ 0.15 M, Na_2_CO_3_ 0.035 M, pH 9.6) and incubated at 4 °C ON. Unbound antigens were removed by washing twice with PBST (PBS and Triton X-100 0.02% (v/v)). The plates were blocked with PBS containing 5% skim milk at 4 °C ON and washed twice with PBST. One hundred µL of blood serum diluted 1:100 (PBS and Triton X-100 0.02% (v/v)) were added and incubated for 2 h at 37 °C, then washed five times and incubated with monoclonal mouse anti-salmon IgM (Ango® FM190AZ5) diluted 1:1000 in blocking solution at 4 °C ON. Then, plates were washed again and incubated with horseradish peroxidase conjugated donkey anti-mouse IgG (Rockland, 610703002) diluted 1:10 000 in PBST. Serum antibody levels were determined using 3,3´5,5´-tetramethylbenzidine as a chromogenic substrate and H2SO4 2 N was used to stop the reaction. Pooled serum obtained from *P. salmonis* experimentally infected fish was used as a positive control. The Absorbance value for each sample was measured at 450 nm. All samples were analyzed in triplicate with negative and positive controls.

### Gene expression analysis of immune markers

We evaluated gene expression of some immune markers in fish at 600 DD post first vaccination in order to evaluate the impact of the vaccine prior to bacterial challenge. Reactions were carried out on a real-time PCR System (Applied Biosystems) using the Terra qPCR Direct TB Green Premix kit (Takara). Total RNA was extracted from 100 mg of head kidney tissue using TRIZOL reagent (Invitrogen), and incubated for 30 min at 37 °C with 20 units of RQ1 RNase-Free DNase (Promega) to remove residual genomic DNA. RNA was purified using the RNeasy mini kit (Qiagen) and RNA concentration was determined as described above. Two μg of total RNA was used for reverse transcription reactions to synthesize single strand cDNA using SuperScript II Reverse Transcriptase and Oligo-dT primers (ThermoFisher Scientific), according to standard procedures. cDNA was diluted to 100 ng and used as the template for qPCR, with primers designed against the following immune markers: Major histocompatibility complex I (*mhc1*), tumor necrosis factor alpha (*tnfα*), cluster of differentiation 8a (*cd8α*)*,* interferon gamma (*ifn*γ), and T cell receptor beta 1 (*trb-I*) as previously published [27–30]. The thermal profile used was 95 °C 10 min, 40 × (95 °C × 30 s, 60 °C × 30 s, 72 °C × 30 s). Relative expression of mRNA was calculated using 2 −ΔΔCT adjusted to primer efficiency [31]. Elongation factor 1-alpha (*EF1a*) was used as housekeeping gene. Primers used are listed in Additional file 2.

### Statistical analysis

Statistical analysis was performed using GraphPad Prism 8 (Graphpad Software, Inc). Fulton´s condition factor was calculated as described above and differences were analyzed using analysis of variance (ANOVA) followed by a Tukey’s multiple comparisons test. Data obtained from gross pathological scoring for Speilberg score, and gene expression was analyzed by Kruskal–Wallis test followed by a Dunn’s post-test for multiple comparisons. Survival curves were analyzed using Kaplan–Meier and group differences were analyzed using Log-rank test. To assess the effectiveness of formulations the relative percent survival (RPS), absolute risk reduction (ARR), and number of animals necessary to treat (NNT) were calculated (all formulas used are described in Additional file 3). Differences in antibodies were calculated using ANOVA and Dunnett’s multiple comparison test. *p* ≤ 0.05 was considered significant and all experiments were performed at least in triplicate.

## Results

### Characterization of *Piscirickettsia salmonis* proteoliposome

Proteoliposomes generated from *P. salmonis* membranes were characterized in terms of size and charge. The average diameter of *P. salmonis* proteoliposomes was shown by dynamic light scattering to be 250.9 ± 13.2 nm (*n* = 10) with a low polydispersity index (PDI) of 0.1349 ± 0.03; indicating the degree of dispersion of sizes. When PDI is 0 all particles are the same size and when the PDI is 1 all particles are different sizes. The zeta-potential, which indicates the charge present in the interface of the particle and the aqueous medium was -37.9 ± 6.3 mV (*n* = 10) (Figures 1B–F).

### Vaccine formulations are safe and do not affect fish weight gain

**Figure 2:**
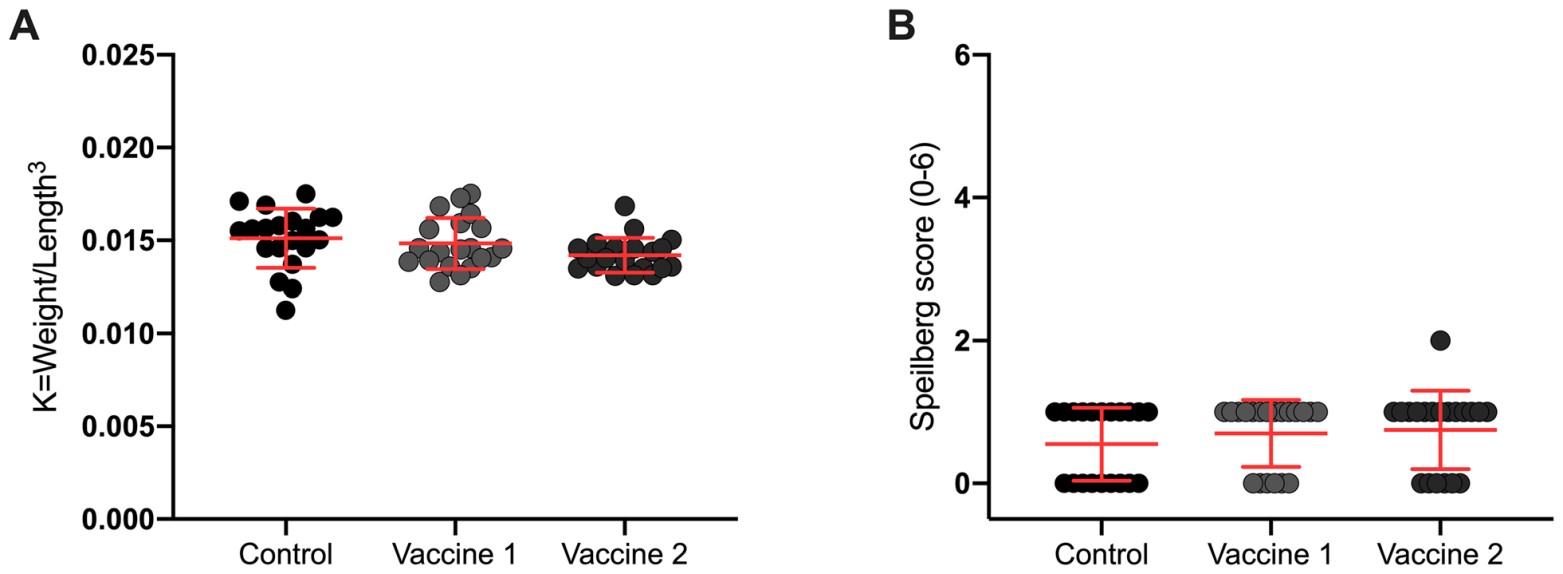
**Safety and adverse effects vaccine evaluation**. **A** Fulton’s condition factor, **B** Speilberg score. Values are represented as dot-plots with mean ± standard deviation. Vaccine 1: 10 µg total protein of *P. salmonis* proteoliposome emulsified with Montanide ISA 760 VG, Vaccine 2: 10 µg total protein of *P. salmonis* proteoliposome emulsified with ISA 763 A VG (Seppic, France).

During the immunization period there were no mortalities. Fish among the different treatment groups were evaluated using Fulton’s condition factor previous to bacterial challenge to evaluate if the vaccines influenced the fitness or condition of fish. Vaccinated fish had no statistically significant differences in Fulton’s condition factor compared to control (*p* ≥ 0.05, ANOVA, Tukey’s multiple comparisons) (Figure 2A). Minimal adverse effects were registered in vaccinated fish during the necropsy and none of the vaccinated groups showed more than 2 points in Speilberg Index (0–6) (vaccine 1: 0.7 ± 0.5; vaccine 2: 0.75 ± 0.6; control: 0.55 ± 0.5); no statistically significant differences were observed between treatments and control (*p* ≥ 0.05; Kruskal–Wallis, Dunn’s multiple comparison) (Figure 2B). Therefore, we can conclude that the vaccines were safe under these experimental conditions.

### *Piscirickettsia salmonis* proteoliposome vaccine induces immunity against *P. salmonis* and modulates gene expression of immunity markers

**Figure 3:**
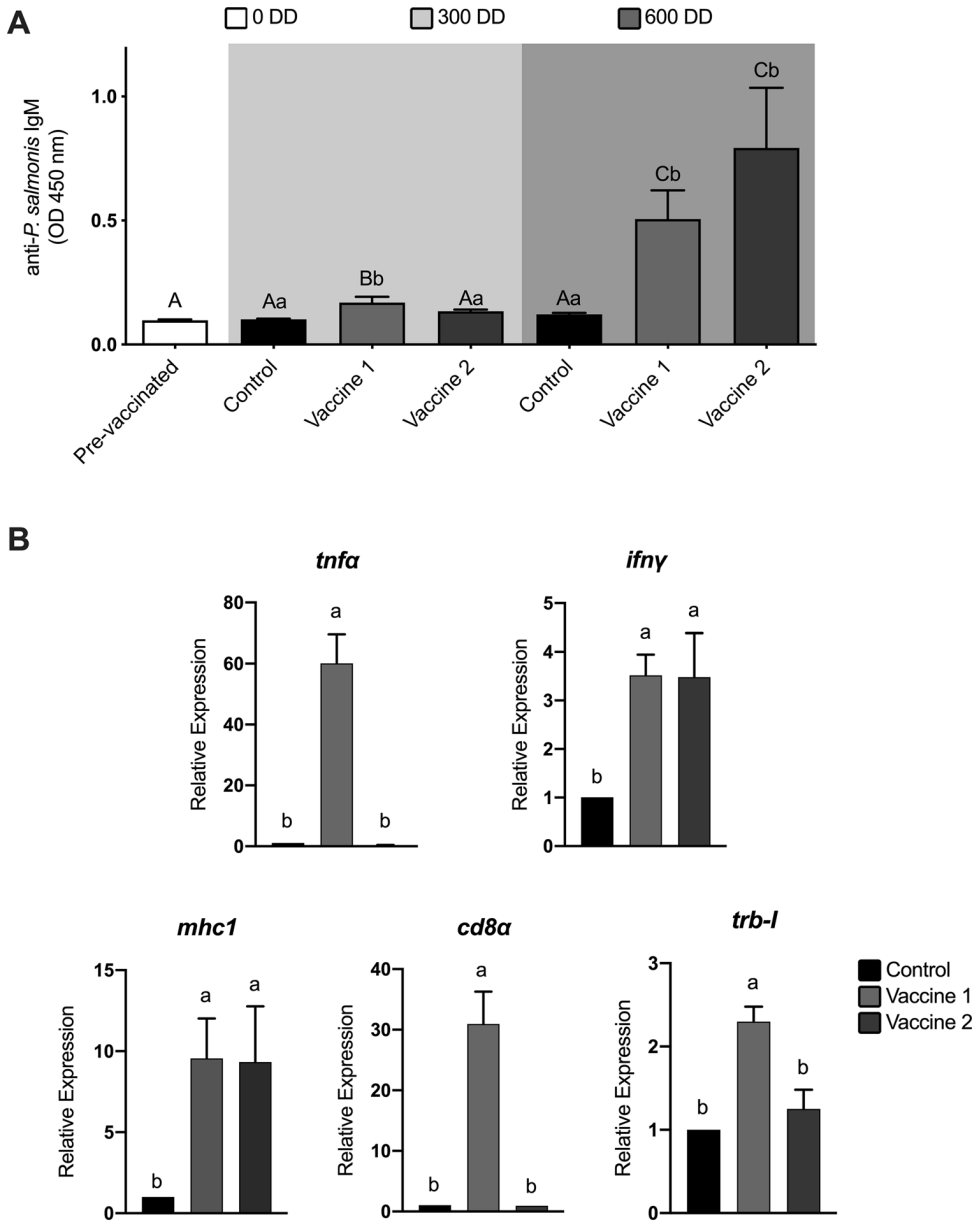
**Effect of vaccines on the level of serum specific anti-*P. salmonis* IgM and gene expression of cell response immunity markers**. **A** The fish were intraperitoneally immunized with two formulations (vaccine 1 or vaccine 2) or a control (injected intraperitoneally with 100 µL of sterile saline). *Piscirickettsia salmonis* specific IgM were measured by ELISA. The data are mean ± SD of 5 fish *per* treatment at each sampling point (pre-vaccination, first immunization, second immunization). ANOVA and subsequent Tukey’s multiple comparisons test. Different capital letters indicate significant differences (*p* ≤ 0.05) between sampling points and lowercase letter indicate significant differences (*p* ≤ 0.05) within the sampling point. **B** Expression of cellular immune response markers analyzed by qPCR. Tumor necrosis factor alpha (*tnfα*), interferon gamma (*ifn*γ), major histocompatibility complex I (*mhc1*), cluster of differentiation 8a (*cd8α*), T cell receptor beta 1 (*trb-I*). The results are the mean ± SEM of 5 individuals, Kruskal–Wallis test followed by a Dunn’s post-test. Different lowercase letters indicate significant differences (*p* ≤ 0.05).

The ability of the vaccines to induce *P. salmonis*-specific IgM in serum was determined by ELISA. No specific antibodies were detected before vaccination or in placebo vaccinated controls. Both vaccines were able to induce a specific anti-*P. salmonis* response (Figure 3A). Specific IgM induced by vaccine 1 was detected earlier than vaccine 2, showing significant differences in relation to the control group at 300 DD post-vaccination (first immunization sampling) (*p* ≤ 0.001). Higher antibody levels were detected at 600 DD post-vaccination (second immunization sampling) for both formulations, showing significant differences in the induction of specific antibodies in relation to the control group (vaccine 1, *p* ≤ 0.05 and vaccine 2, *p* ≤ 0.001). No difference between the vaccines were detected at 600 DD. Significant increases in specific IgM levels were observed for vaccine 1 and vaccine 2 (four and six-fold compared to control and pre-vaccination samples); confirming that a proteoliposome based vaccine can induce an immune response in *O. mykiss*.

The relative expression of some immune markers at 600 DD post-vaccination was measured to determine the ability of the vaccines to induce a cellular immune response. Transcripts were grouped as innate response (*tnfα, ifn*γ) and cell-mediated immunity markers (*mhc1*, *cd8α*, *trb-I*). The transcriptional profile for vaccine 1 showed all transcripts were upregulated compared to control; where the transcriptional profile for vaccine 2 showed increases for *mhc1* and *ifn*γ only (Figure 3B).

### Piscirickettsia salmonis proteoliposome vaccine protects against challenge

**Figure 4:**
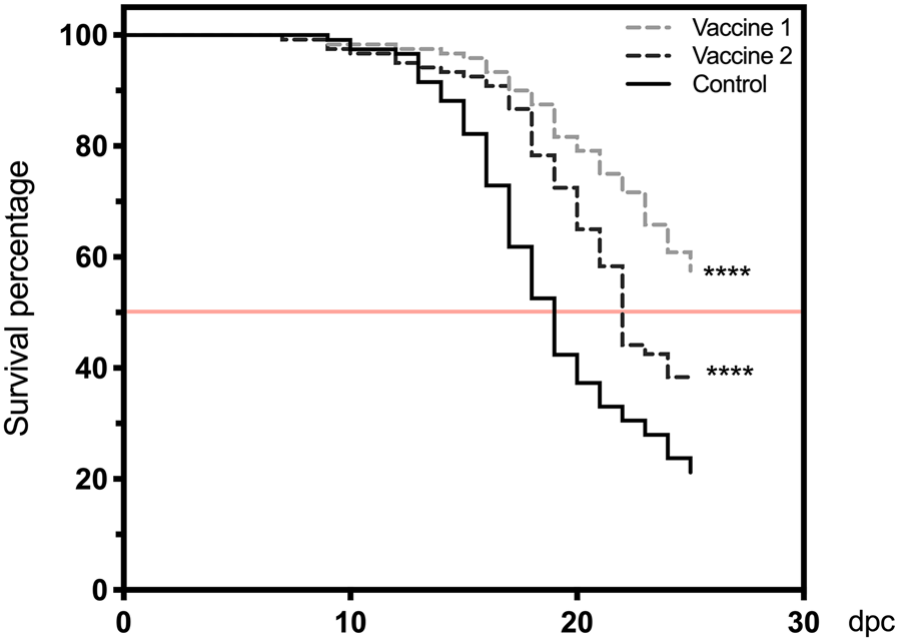
**Effect of intraperitoneal vaccination on survival of fish infected with a lethal dose of*Piscirickettsia salmonis.*** Fish subjected to vaccination and challenged by intraperitoneal inoculation against *P. salmonis*. Survival was monitored on a daily basis. Kaplan–Meier and subsequent survival curves comparison by Log-rank test. Asterisks show statistically significant differences between groups and denote: **** *p* ≤ 0.0001. Red light line shows 50% cumulative survival.

The efficacy of the vaccines was determined by challenging fish at 300 DD post-second immunization with a lethal dose of *P. salmonis* by i.p. inoculation, previously determined by the LD_50_ challenge experiment (Additional file 1). Mortality was monitored on a daily basis. Both vaccines conferred significant protection compared to the non-immunized control (Log-rank test, *p* ≤ 0.0001). Vaccine 1 had a better final survival percentage than vaccine 2 (57.5 and 37.5% respectively, Log-rank test, *p* ≤ 0.0010) (Figure 4). The relative percentage survival (RPS) and the absolute risk reduction (ARR) were calculated at the end of the trial. The RPS and ARR estimate the reduction of risk of death by *P. salmonis* in vaccinated vs. non vaccinated fish (in relative terms for RPS and in absolute terms for ARR). We also calculated the number of animals necessary to treat (NNT), a parameter that indicates the number of animals that must receive the treatment (vaccine) in order for one animal to survive in the evaluated time interval (25 days). Vaccine 1 had an RPS of 46.06%, an ARR of 36.31% and NNT = 3; while vaccine 2 had an RPS of 20.68%, an ARR of 16.31% and NNT = 7. The results indicate that both vaccines are able to protect fish, but vaccine 1 showed better efficacy than vaccine 2 (Additional file 3).

## Discussion

The strategy of developing proteoliposome from a bacterial substrate has been explored primarily in murine models with projected use in humans [20, 32–34]. In recent years there has been an increase in publications related to the use of liposomes, and micro- and nanoparticles for the development of new vaccines in aquaculture [35–37]. The use of proteoliposomes generated from *P. salmonis* membranes is a novel approach to generate a vaccine without antigen degradation due to heat- and formalin-inactivated bacteria. Proteoliposome formulations contain microbial- or pathogen-associated molecular patterns (MAMPs or PAMPs) that act as immunopotentiators and also exerts delivery system ability as well [23]. The use of proteoliposomes as vaccines for salmonids has not been addressed until now.

The physicochemical characteristics that can affect the performance of a vaccine are size, shape, surface load, hydrophobic and hydrophilic capacity, as well as the ability to interact with host receptors [38]. Many of these factors are related to the ability to interact with antigen-presenting cells (APCs) and the correct induction of a protective immune response [39]. While there is not much literature regarding particle size and immune response in teleosts, size is described as an important factor; APCs and B-lymphocytes are capable of phagocytizing particles of at least 1 µm [40]. In turbot (*Scophtalmus maximus*) it has been determined that the maximum particle size to reach a secondary lymphoid organ after an intraperitoneal inoculation is less than 4 µm [41]. This information is relevant since if the antigen is not capable of reaching secondary lymphoid organs in sufficient quantities it will be ignored immunologically [42]. The size obtained during the *P. salmonis* proteoliposomes characterization was less than 1 µm on average (250.9 ± 13.2 nm) and could be classified as nanoparticles [36]; making them small enough to reach secondary lymphoid organs in teleosts.

Results obtained when quantifying the zeta potential are directly related to the source of the lipid used [43]. In this case the membranes were obtained directly from *P. salmonis* presenting anionic charge on the surface with zeta potential values below −30 mV (−37.9 ± 6.3 mV). This indicates that the vaccines present good stability and dispersion [36]. Ionized liposomes (either positively or negatively) have a better adjuvant performance than neutral ones [44]. The internalization of ionized particles by APCs increases up to 1.3 times in relation to neutral ones [45] and it has been described that scavenger-type receptors present in APC are able to recognize anionic particles, facilitating their internalization [46]. Finally, some studies have shown that anionic liposomes induce a Th1 biased cellular immune response: a desirable characteristic for a vaccine designed against an intracellular pathogen like *P. salmonis* [44, 47]. Although much of this information comes from studies conducted in mammals, results obtained in trout macrophages and zebrafish using anionic liposomes have shown increased internalization [48, 49], suggesting a conserved mechanism.

There are safety concerns when using injectable vaccines based on oil adjuvants, since adverse effects can occur. No reduction in growth was observed in the present study at 300 DD post-immunization using Fulton’s condition factor as a measure; although there are studies describing growth reduction with oil-based vaccines in salmonids [50, 51]. Macroscopic observations of vaccinated and control fish revealed mild changes in vaccinated groups during post-mortem examination without significant differences in relation to control using Speilberg index as an evaluation tool to address adverse effects. Therefore, we can say that both vaccines are safe.

Bacterin based vaccines will, in general, elicit an immune response biased toward humoral immunity with a lesser induction of cell-mediated immune response [52]. Antibodies will attach to surface antigens of the pathogen resulting in opsonization and phagocytosis [53, 54]. *P. salmonis* is an intracellular pathogen, therefore these functions are useful during early stages of infection, from port of entry (gills, skin, and gut) and transport to primary or secondary multiplication sites [55]. There is no information on how *P. salmonis* disseminates in the host. If an extracellular pathway is not used, the usefulness of antibodies in limiting the infection would be reduced. It is known that there is a correlation between antibody titers and protection against mortality in *P. salmonis* infection [55, 56]. Consequently the induction of antibodies is a desired effect. Immune response elicited in terms of specific anti-*P. salmonis* IgM in vaccinated groups showed a significant induction at 300 and 600 DD after first immunization for vaccine 1 compared to control. Vaccine 2 only showed a significant induction at 600 DD after first immunization compared to control. The second immunization enhance the magnitude of antibody induction showing a 4 and sixfold anti-*P. salmonis* IgM compared to control without significant differences between vaccines (Figure 3A). Injected anti-SRS vaccines are able to induce specific IgM antibodies. When the concentration of antibodies is below 2000 pg/mL in farmed salmonids a window of susceptibility to SRS infection was observed, suggesting a close association between antibody levels and protection [56].

Teleosts have a specific cell-mediated immunity characterized by antigen presentation by MHC-I to CD8 T lymphocytes [56–59]. Therefore, we evaluated the gene expression of markers associated with the stimulation of a cellular immune response using head kidney. To analyze the inflammatory response, we measured the expression of *tnfα*. *tnfα* was strongly upregulated in fish receiving vaccine 1, but no induction was observed in vaccine 2. Fish immunized with vaccine 1 showed upregulation of *mhc1*, *cd8a*, *ifn*γ, and *trb-I* suggesting that vaccine 1 is promoting a CD8 T cell mediated response. Vaccine 2 induced an upregulation of a reduced number of transcripts different from control, when compared to vaccine 1. Unlike the vaccines developed for this study which are proteoliposome based, *P. salmonis* bacterin based vaccines in *Salmo salar*, induce an upregulation of *mhc1* but downregulation of *cd8* suggesting that bacterin based vaccines support a CD4 T cell response and are unable to induce a CD8 T cell immune response [60]. Transcriptional results using the vaccines from this study suggest that a cellular response is being induced by the *P. salmonis* proteoliposome based vaccine.

Efficacy of vaccines against SRS are variable and depend on the type of vaccine, route of immunization and the lethality of *P. salmonis* strain used in the challenge [55]. The majority of studies available use *Salmo salar* or *O. kisutch* and showed RPS rates ranging from 10 to 90% [4, 61–66]. There is only one study that evaluates vaccine efficacy in *O. mykiss*. Smith et al., determined the efficacy of an injectable *P. salmonis* vaccine made from formalin-killed bacterin. The cumulative mortality was lower than nonvaccinated fish. However, lethality of the *P. salmonis* challenge strain was not strong enough because mortality in the infected nonvaccinated group was low (20%) [67].

In this study, the first challenged fish died 7 days post-challenge (DPC), mortality then increased in all groups from day 10 onwards. These results are in agreement with the previously reported incubation period for *P. salmonis* LF89 i.p. infection of 10–20 days [60, 65]. Cumulative mortality of the control group during the challenge was 78.8%. The lowest mortality during LD50 determination was 96.7%, indicating the high virulence of the bacterial isolate used in this study. Both vaccine formulations successfully conferred protection against SRS, as shown by the survival curve analysis (vaccine 1, *p* ≤ 0.001; vaccine 2, *p* ≤ 0.0001). The better cellular immune response profile exhibited by vaccine 1 may explain why vaccine 1 provided better protection than vaccine 2. It is possible that the survival percentages for vaccine 1 (57.5%) and vaccine 2 (37.5%) are underestimated due to the lethality of the *P. salmonis* challenge and the inoculation route used.

Results obtained in the current study demonstrate that immunization with *P. salmonis* proteoliposomes induces a significant protection against challenge with a lethal *P. salmonis* isolate and is able to induce specific anti-*P. salmonis* IgM after a second dose with a transcriptional profile that suggests a CD8 T cell-mediated immunity. Although this first prototype shows promising results, survival due to the vaccine could be improved with the addition of recombinant antigens or by increasing the antigen concentration. Future efforts will focus on increasing the efficacy of the vaccine formulation as well as further evaluation of the transcriptional profiles of the cellular response to vaccination. This is only the second work published evaluating the efficacy of a *P. salmonis* vaccine on *O. mykiss.* SRS is the leading cause of death in trout farming, therefore further study is important. The generation of proteoliposomes to produce antigens for vaccines is potentially applicable to other pathogens important to fish farming. It is also possible to include antigens from other pathogens of interest to create multivalent vaccines.

## Supplementary Information

**Additional file 1**:** Cumulative mortality (%) in*Oncorhynchus mykiss* after*Piscirickettsia salmonis* challenge, LD50 determination**. Dilutions used in the challenge 1/10, 1/100, 1/1000, 1/10 000.

**Additional file 2**:** List of primers used for gene expression studies (previously published)**.

**Additional file 3:** **Efficacy indicators for** ***P. salmonis*** **vaccines and mathematical formulas.**

## Data Availability

The datasets during and/or analyzed during the current study are available from the corresponding author on reasonable request.

## Abbreviations

SRS: salmonid rickettsial septicaemia
IgM: immunoglobulin M
T CD8: CD8+ T cells
SAG: Servicio Agrícola Ganadero
DD: degree days
ON: overnight
*mhc1*: major histocompatibility complex I
*tnfα*: tumor necrosis factor alpha
*cd8α*: cluster of differentiation 8a
*ifn*γ: interferon gamma
*trb-I*: T cell receptor beta 1
*EF1a*: T cell receptor beta 1
RPS: Relative percent survival
ARR: absolute risk reduction
NNT: number of animals necessary to treat
PDI: polydispersity index
i.p: intra-peritoneal
