# Supplementary figures and images for: Effectiveness of a proteoliposome-based vaccine against salmonid rickettsial septicaemia in Oncorhynchus mykiss
Source: Vet Res. 2021 Aug 23;52:111. doi: 10.1186/s13567-021-00982-2 (PMC8382212; doi:10.1186/s13567-021-00982-2)

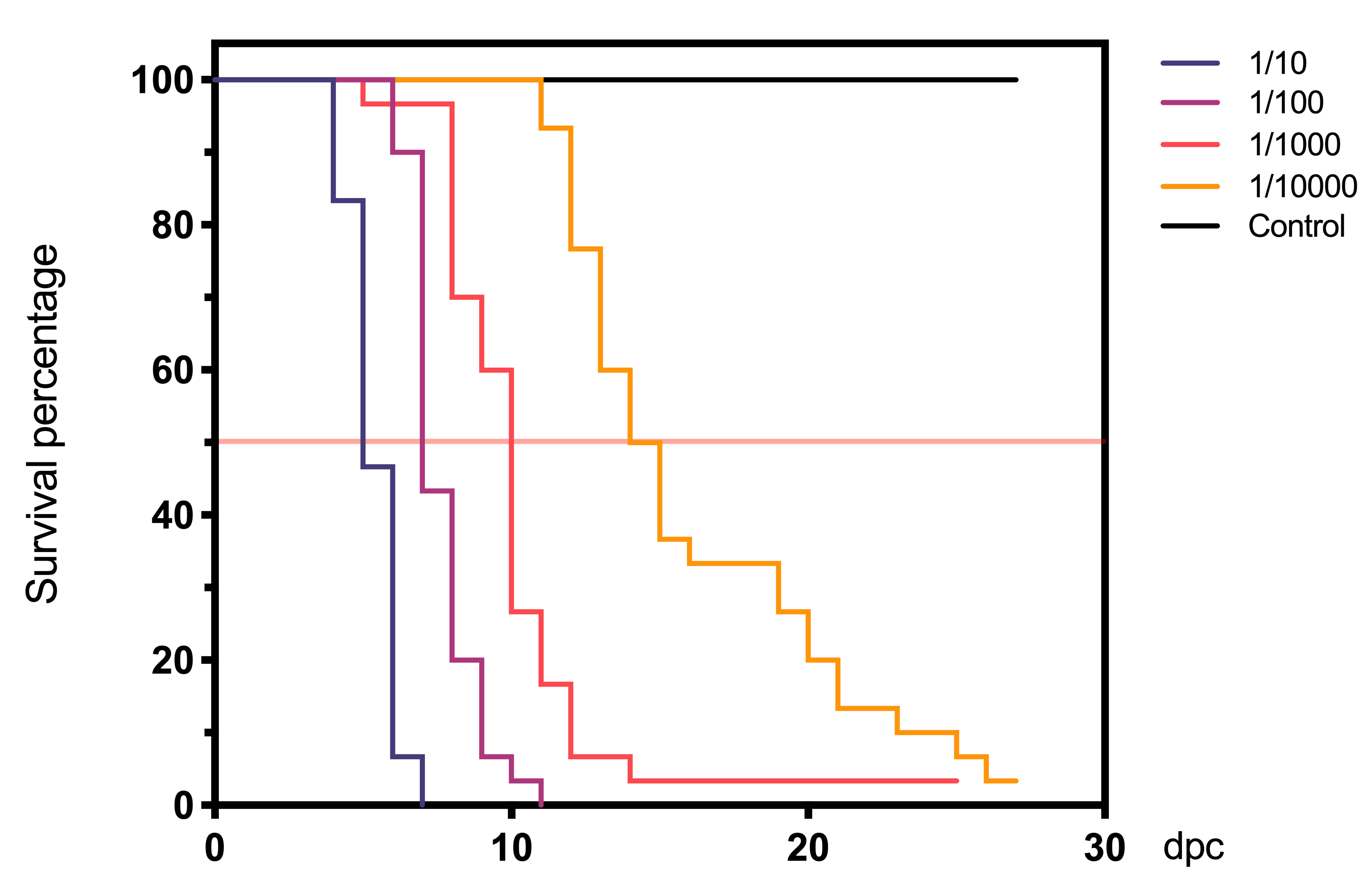

Supplement: Supplementary file 1 — Additional file 1: Cumulative mortality (%) inOncorhynchus mykiss afterPiscirickettsia salmonis challenge, LD50 determination. Dilutions used in the challenge 1/10, 1/100, 1/1000, 1/10 000. [file 13567_2021_982_MOESM1_ESM.tiff]
