# Supplementary material for: Effectiveness of a proteoliposome-based vaccine against salmonid rickettsial septicaemia in Oncorhynchus mykiss
Source: Vet Res. 2021 Aug 23;52:111. doi: 10.1186/s13567-021-00982-2 (PMC8382212; doi:10.1186/s13567-021-00982-2)
Supplement: Supplementary file 2 — Additional file 2: List of primers used for gene expression studies (previously published). [file 13567_2021_982_MOESM2_ESM.docx]

**Additional file 2.** **List of primers used for gene expression studies (previously published).**

| **Gene name** | **Forward sequence 5´-3´** | **Reverse sequence 5´-3´** | **Genbank accession no.** | **Reference** |
| --- | --- | --- | --- | --- |
| *MHC Class Ib* | TCCCTCCCTCAGTGTCT | GGGTAGAAACCTGTAGCGTG | AY523661 | [29] |
| *tnfα* | GGGGACAAACTGTGGACTGA | GAAGTTCTTGCCCTGCTCTG | AJ277604 | [28] |
| *cd8α* | ACACCAATGACCACAACCATAGAG | GGGTCCACCTTTCCCACTTT | AF178054 | [28] |
| *ifnγ* | AAGGGCTGTGATGTGTTTCTG | TGTACTGAGCGGCATTACTCC | NM_001124620 | [29] |
| *trb-I* | GTCTTCTGGCAAGTCAACAATGT | GTAAAAGCTGACAATGCAGGTGA | EU072699 | [27] |
| *EF1a* | ACCCTCCTCTTGGTCGTTTC | TGATGACACCAACAGCAACA | AF498320 | [30] |
|  |  |  |  |  |
