# Supplementary material for: Effectiveness of a proteoliposome-based vaccine against salmonid rickettsial septicaemia in Oncorhynchus mykiss
Source: Vet Res. 2021 Aug 23;52:111. doi: 10.1186/s13567-021-00982-2 (PMC8382212; doi:10.1186/s13567-021-00982-2)
Supplement: Supplementary file 3 — Additional file 3: Efficacy indicators for P. salmonis vaccines and mathematical formulas. [file 13567_2021_982_MOESM3_ESM.docx]

**Additional file 3. Efficacy indicators for *P. salmonis* vaccines and mathematical formulas**

| **Indicators** | **Vaccine 1** | **Vaccine 2** | **Control** |
| --- | --- | --- | --- |
| **% Mortality** | 42.5 | 62.5 | 78.8 |
| **% RPS** | 46.06 | 20.68 |  |
| **% ARR** | 36.31 | 16.31 |  |
| **CI 95% ARR** | 24.80 to 47.83 | 4.94 to 27.69 |  |
| **NNT** | 3 | 7 |  |
| **CI 95% NNT** | 2.1 to 4.0 | 3.6 to 20.2 |  |

Indicators calculated in relation to control group.

RPS: Relative percentage survival, ARR: Absolute risk reduction, NNT: Number necessary to treat, CI: Confidence interval.

| **Test name** | **Formula** |
| --- | --- |
| Relative Percent Survival (RPS) | $1- \frac{Cumulative mortality of treated group}{Cumulative mortality of control group non treated} x 100$ |
| Absolut Risk Reduction (ARR) | $\left( \left( \frac{Cummulative mortality of treated group}{Total of treated group} \right)-\left( \frac{Cummulative mortality of non treated group}{Total of non treated group} \right) \right)x 100$ |
| Number of animals necessary to treat (NNT) | $\left( \frac{1}{ARR} \right)*100$ |

**Mathematical formulas used to evaluate effectiveness parameters**
